# Influence of unidirectional polishing on the formation of laser-induced periodic surface structures on steel

**DOI:** 10.1038/s41598-025-19844-4

**Published:** 2025-10-14

**Authors:** Ona Balachninaitė, Eugenijus Gaižauskas, Gytis Zaremba, Domas Paipulas

**Affiliations:** Faculty of Physics, Laser Research Center, Vilnius University, Saulėtekio Ave. 10, Vilnius, Lithuania

**Keywords:** Engineering, Materials science, Surface patterning

## Abstract

We have performed a detailed analysis of the dependence of the regularity of laser-induced periodic surface structures (LIPSS) on the surfaces of steel samples that have been ground (polished) to achieve unidirectional surface roughness. Two low spatial-frequency LIPSS data sets were created by scanning the steel surface with femtosecond laser pulses using field polarisation perpendicular and parallel to the direction of the initial linearity of the surface roughness. The images were analysed by SEM, and a database of LIPSS characteristics was created using a digital image processing module. It was found that the regularity of the LIPSS depends on the polarisation direction of the scanning beam with respect to the linearly polished surface. A statistically significant result was evaluated: scanning perpendicular to the direction of the initial roughness leads to more regular low spatial frequency LIPSS. Compatibility with electromagnetic scattering theories is demonstrated.

## Introduction

Laser-induced periodic surface structures (LIPSS, also known as ripples), first demonstrated on semiconductor surfaces by Birnbaum^1^, have drawn a lot of attention over the past twenty years because of advancements in the production of ultra-short pulsed lasers and the numerous technological applications for surface modification to control their chemical, optical and mechanical characteristics^2,3^.

It is important to note that despite the significant theoretical and experimental efforts aimed at elucidating the mechanisms underlying LIPSS formation (we refer here to recently published reviews^4–10^, where comprehensive state-of-the-art analysis is given), explaining the creation of these periodic micro- and nanostructures is more complicated than producing them.

Indeed, it is well-established that both the interference of the incoming and defect-scattered light and the interference with surface plasmon-polariton (SPP) waves are responsible for the periodic temperature modulation on the surface. Nevertheless, the majority of the existing models (see e.g.^11–17^), investigate only the periodic modulation of the electron and ion temperatures, leaving unanswered the more complicated question of how the induced inhomogeneous temperature distribution causes the periodically modulated surface profile.

In fact, practical applications of LIPSS technology require a larger area to be covered by periodic structures^6,7,18^. Many applications, such as enhancing light absorption in solar cells^19^, controlling surface wetting^20^, and forming multifunctional surfaces^21^, require LIPSS to be present over a large area of the material. And there is even less clarity on an important issue: the development of LIPSS over a large area. Generating LIPSS over large areas is difficult because it requires maintaining uniformity and consistency across the entire surface. Although some theoretical models exist, a comprehensive, predictive understanding of LIPSS formation mechanisms for large-area processing is lacking^6^. Technical difficulties in scaling LIPSS and the current lack of reliable, uniform fabrication strategies over large areas are also issues^7,22^. Detailed studies of the regularity and physical properties of the formed LIPSS, obtained by scanning surfaces with a laser beam, are therefore of great importance. Recently, variations of the generated LIPSS quality on metal surface morphology^23^, surface tilt angle^24^, as well as LIPSS self-alignment and the possibility to control the half-periodic mismatched optical enhancement through designed laser scanning strategies^25^ have been found. Noteworthy is the strong impact of linear polishing on the generation of low spatial frequency LIPSS (LSFL) demonstrated in^26^, where it was shown that for smooth surfaces the polishing direction has no influence on the LSFL orientation whereas for a surface roughness $>0.187$ $\upmu$m the LFSL direction is driven by the polishing direction and LSFL generated with a low fluence are more attracted by the surface polishing. On the other hand, in a similar study^27^, no apparent effects of the LIPSS spatial periods and orientation on the initial surface conditions were reported.

Several aspects crucial to maintain consistent LIPSS formation can be identified for these contradictory conclusions: irregular beam profile and energy instability, superficial heterogeneity of the surface, thin oxide layer or impurities affecting energy absorption, environmental conditions, etc., which may result in variations in the structure of the LIPSS formed depending, e.g., on the location of the measurement. In our previous work^28^, it was found that higher-quality LIPSS tended to form on samples with a polished surface. However, a recent study by^29^ found that even the crystallographic orientation of the grains has an impact on the formation of the low-spatial-frequency LIPSS (LSFL), leading to changes in the orientation of the grooves. Therefore, an interesting issue that requires more attention and investigation is the influence of the initial roughness directionality on the LIPSS regularity.

The quality of the LIPSS formed in^25–27^ was determined by comparing the SEM images without the use of quantitative parameters and, therefore, lacks objectivity. In recent paper^30^, it was shown experimentally that the magnitude and preferred direction of the roughness of the substrate affect the coherence and orientation of the LIPSS. It was shown that a roughness direction perpendicular to the laser polarization enhances LIPSS regularity, reducing DLOA values, while a parallel orientation has the opposite effect. In our work, since the ideal unidirectional roughness is not easy to design, we performed more experiments to make the result statistically reliable. In addition, we have not only found a preferred direction of the unidirectional roughness of the substrate with respect to the laser light polarisation to form LIPSS of higher linear regularity but also explained why this result is obtained on the basis of electromagnetic scattering theories.

We aimed to study the dependence of LIPSS morphology on steel surface samples, initially possessing some directionality of roughness produced during linear (unidirectional) surface grinding. Specifically, we investigate the LIPSS regularity parameter *R* defined in our previous paper^28^, using two beam scanning directions (perpendicularly and parallel to the predominant surface directionality). The detailed study of the scanning beam overlap, the power of the femtosecond laser pulses, and many LIPSS images obtained under different conditions allow us to draw statistically significant conclusions on the question of interest.

## Experimental setup for laser fabrication of LIPSS

The femtosecond ($\sim$ 200 fs) laser system (diode-pumped femtosecond laser with an integrated oscillator and amplifier) used in this experiment was a Pharos Yb:KGW laser (Light Conversion Ltd., Lithuania) operating at a wavelength of 1030 nm, with an average output power of up to 6 W. An integrated pulse picker allowed pulse-on-demand operation and a variable repetition rate from single-shot pulses up to 1 MHz. For the formation of laser-induced periodic surface structures, the repetition rate was fixed at 6 kHz, and the average output power was fixed at 750 mW. An additional attenuator, consisting of a half-wave plate and a thin film polariser, was used to vary the incident laser power on the sample from 40 to 110 mW (which corresponds to the energy fluence from 0.9 to 2.6 J/cm^2^, respectively). The focal length of the f-theta lens was 100 mm. The diameter of the laser beam at the focal point was determined to be ($30 \pm 2$) $\upmu$m using Liu’s method^31^.

**Fig. 1:**
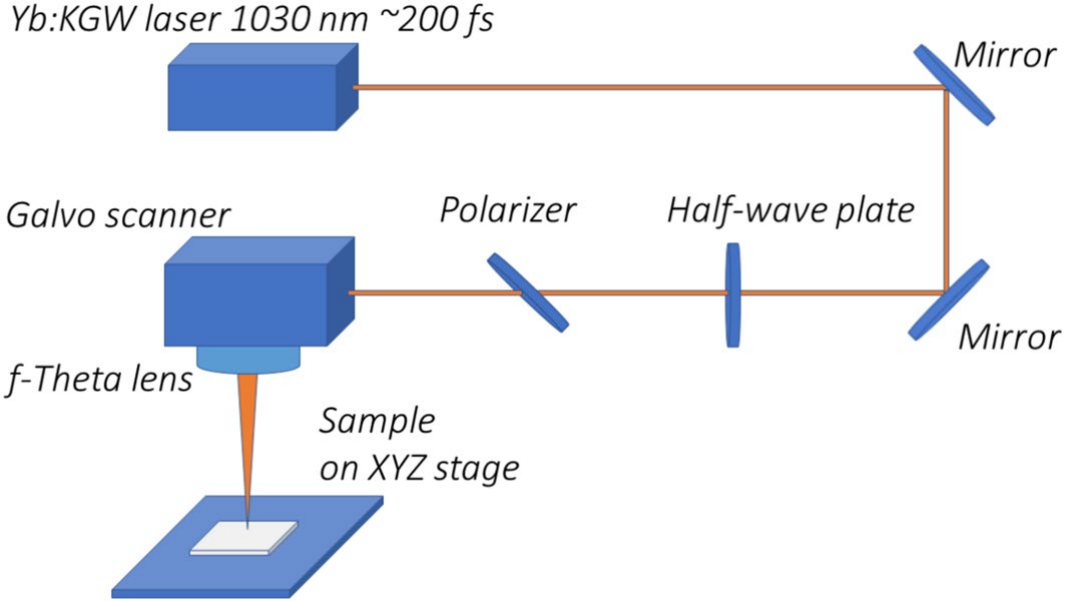
Experimental setup for LIPSS formation.

*XYZ* translation stages were used for precise beam positioning (see Fig. 1). The system was controlled by DMC software (1.4.71 version) (Direct Machining Control Ltd.). This program was used to control the galvo scanner and pulse picker of the laser. Stainless steel (304S) with the initial surface roughness Ra of ($260 \pm 5$ nm was chosen for the experiments. The surface roughness Ra (the arithmetic average of the absolute values of the roughness profile ordinates) was measured using an Olympus OLS5100 laser confocal microscope. Parallel lines were written with a wide spacing so that the irradiated areas would not intersect. The spatial overlap between subsequent laser beams along the X-axis was set by the scanning speed controlled by the software. The beam-to-beam overlap (OV) expressed as a percentage can be calculated as a function of the center-to-center distance between two consecutive laser beams on the surface (x) and the diameter of the laser beam (d) using the expression

1 $$OV = \left( {1 - \frac{x}{d}} \right) \times 100\%$$

**Fig. 2:**
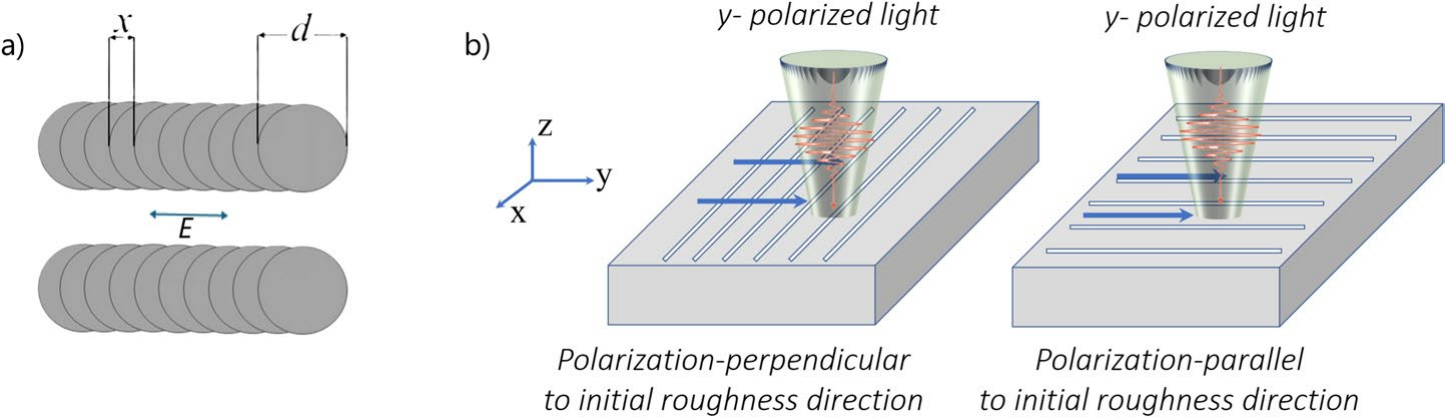
Experimental geometry used to form LIPSS: (**a**) illustration of the scanning algorithm; d is the diameter of the laser beam, and x is the distance between the subsequent laser beams. The parallel lines are not overlapping. The double arrow between the lines shows the polarisation state of the laser beam used; (**b**) geometry for perpendicular (left) and parallel (right) to the initial roughness directionality polarisation of the laser beam. White lines on the surfaces correspond to the roughness direction. Blue arrows show the scanning direction.

A schematic of the algorithm used is shown in Fig. 2. In all experiments, the laser polarisation was aligned parallel to the scanning direction to ensure consistent LIPSS orientation perpendicular to the scan path, which is known to improve pattern regularity and surface uniformity during raster scanning^32^.

Note that the steel samples were polished (ground) to create the initial roughness linearity, and LIPSS was formed in two scan directions for each of them: parallel and perpendicular to the initial roughness directionality.

## LIPSS characterisation

To achieve the objectives set out in the Introduction, different scanning directions, parallel or perpendicular to the prevailing surface roughness grooves, were chosen in our experiments. Note that in both scanning geometries (shown in the Fig. 2), the direction of E-Field polarisation relative to the scanning direction remains the same, i.e. parallel or perpendicular to the initial roughness directionality.

**Fig. 3:**
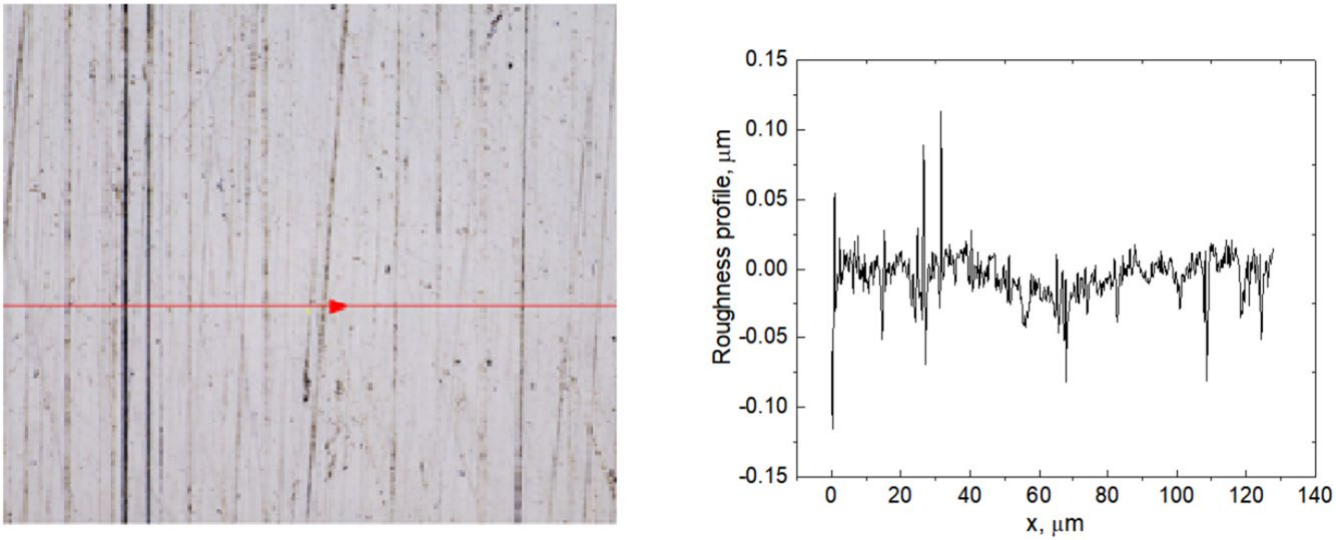
Microscope image (scale: 128 × 128 µm^2^) (left) with the corresponding roughness profile (right) of the initial (untreated by laser) surface of the sample linearly polished at 2500 (10 µm).

**Fig. 4:**
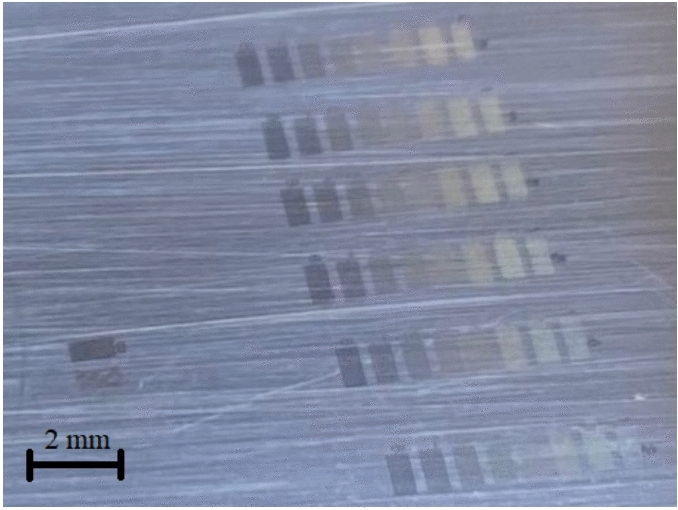
Picture of the prepared (strait-polished) steel surface used in experiments for LIPSS regularity analysis. Places of the formed LIPSS are seen on the right side of the picture.

The stainless-steel sample was pretreated by linear polishing with abrasive. The grain size used was 10 µm (abrasive sheets, grit grade 2500). A sample with a surface roughness between Rz = 0.353 µm and Ra = 0.016 µm was prepared after this process, with Ra being the average roughness of the surface and Rz the value between the highest peak and the lowest valley. The initial roughness of the polished sample was characterised before irradiation using Olympus OLS5100 laser confocal microscope. A microscope image with the corresponding roughness profile of the strait-polished surface of the sample is shown in Fig. 3. The strait-polished steel surface prepared for the experiments is shown in Fig. 4 together with some of the formed LIPSS, seen on the right side of the image.

### Surface roughness characterisation

The “Amount” column marks the ratio of the sum of the real histogram values from the “center − std” to the “center + std” around the main Gaussian peak, to the total sum of the histogram values. In fact, the normalized values here indicate the prevalence of structures oriented in the primary direction, and some proportionality to those in the “Dispersion” column can be observed in case of the small to moderate salience of the main Gaussian peak in the histogram. The ’Goodness’ column reports the goodness of Gaussian function fitting (“1” is good, “0” is bad) on the directionality histogram using the Simplex algorithm and measures the confidence in the identified dominant orientation and reflects the sharpness and distinctness of the peak in the orientation histogram. Together, the last two parameters (“Amount” and “Goodness”) provide a quantitative basis for evaluating the regularity and quality of LIPSS and serve for the assessments of the histogram parameters of interest: “Direction” and “Dispersion”.

The results in Table 1 show that the surface roughness of the individual parts of the prepared steel sheet varies from place to place, and the percentage of structures oriented in the dominant direction is not high, as indicated by the “Amount” parameter. In addition, the unavoidable influence of beam intensity fluctuations when using a femtosecond laser source for LIPSS formation should be considered. Therefore, in order to answer the question of interest regarding the influence of surface roughness on the quality of LIPSS, it is necessary to collect sufficient data for the results to be statistically significant.

**Table 1:** Examples of directionality analysis on the individual locations of the ground steel sheet.

Location	Direction (°)	Dispersion (°)	Amount (*a*.*u*.)	Goodness (*a*.*u*.)
1	107.62	1.61	0.04	0.66
2	111.00	7.74	0.15	0.14
3	85.47	2.50	0.06	0.66
4	57.77	12.16	0.26	0.10
5	85.36	1.95	0.05	0.87
6	85.62	2.08	0.06	0.75

**Fig. 5:**
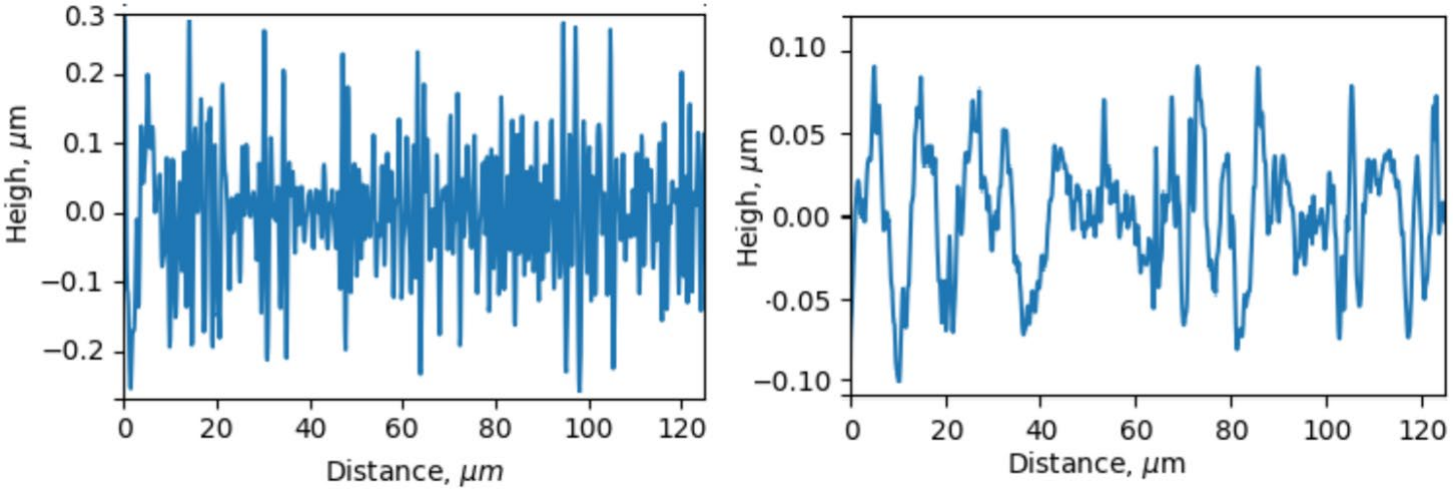
Typical roughness profiles of the steel surface perpendicular (left) and parallel (right) to the non-irradiated (ground only) linearity of the surface roughness.

Typical initial roughness profiles of the steel surface measured in two directions, perpendicular and parallel to the orientation of the unidirectional polishing, are shown in Fig. 5. It is seen from this picture that the roughness profile perpendicular to roughness directionality is characterised by a considerably higher depth of grooves compared to those measured in a parallel direction. Numerical evaluations for one of the key metrics of surface finish quality RMS (Root Mean Square) in two directions give 0.11 $\upmu \text{m}$ and 0.04 $\upmu \text{m}$, respectively, with significantly longer correlation length for the first one. The estimated roughness parameters will be used further when analyzing the experimental results in the Discussion section.

### LIPSS regularity evaluation

Usually, LIPSS regularity is characterised by the dispersion of the LIPSS orientation angle (DLOA)^33^. If the DLOA is less than 10 degrees, the LIPSS is called highly regular (HR-LIPSS). However, as far as dispersion angles are concerned, the corresponding values in the “Amount” column must be emphasized. As noted in the previous subsection, a small value here indicates a small fraction of the preferred orientation in the histogram.

**Fig. 6:**
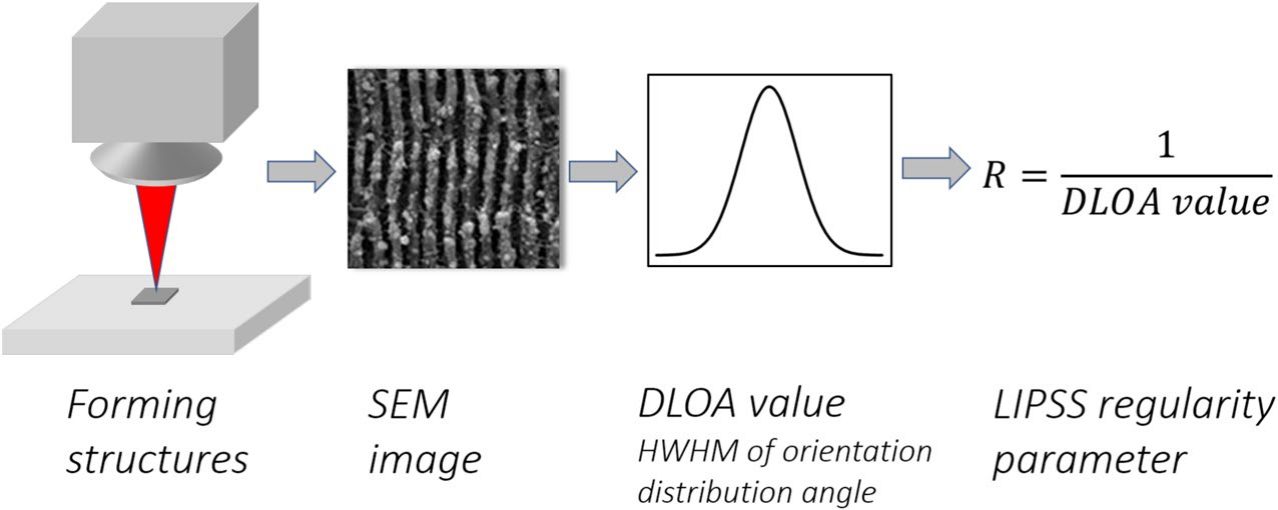
Sketch of evaluation steps of the regularity parameter *R* from SEM image of LIPSS.

A scanning electron microscope (SEM) was used to capture high-resolution images of the fabricated structures as demonstrated, and following the method used in our previous paper^28^, the regularity of the LIPSS was characterised by the parameter *R*, defined as the reciprocal of the DLOA value. The evaluation steps for this parameter (formation of structures, acquisition of SEM image, evaluation of DLOA and R) are shown in the Fig. 6.

**Fig. 7:**
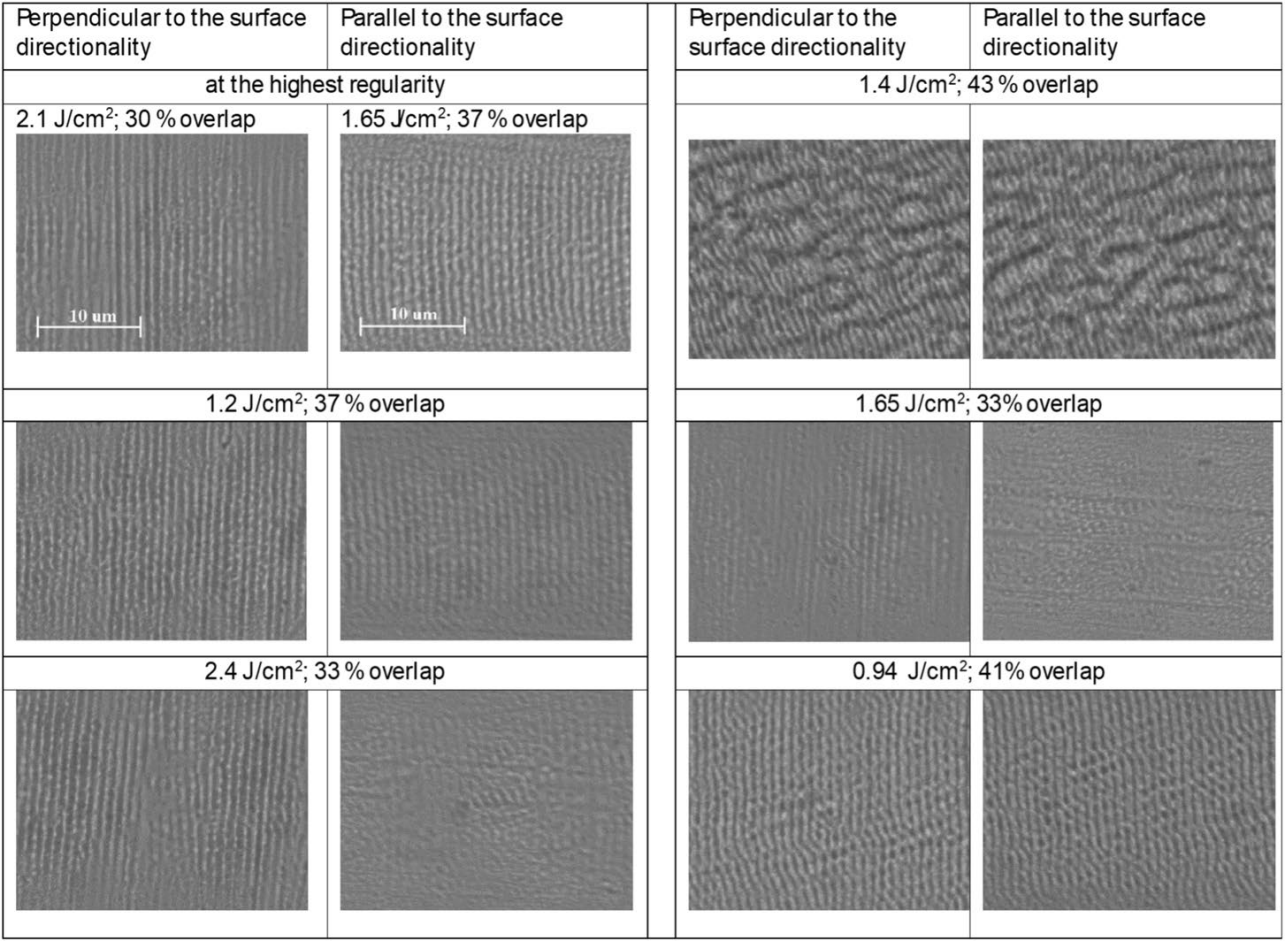
Some of SEM images of LIPSS (at different experimental parameters) formed using parallel and perpendicular scanning directions (or beam polarisation) to the initial surface roughness directionality.

It should be emphasised that such an evaluation method requires surface imaging and digital processing and does not consider the depth of roughness and ripples. Nevertheless, this procedure is rather accurate for regularity evaluation. The LIPSS regularity values were obtained by averaging the results of SEM images taken at 6 different sample locations. In total, more than 600 SEM images were processed. Some of the typical SEM images of LIPSS formed using parallel and perpendicular scanning directions to the initial surface roughness directionality are shown in Fig. 7. The SEM images were all obtained with the same magnification. In the case of perpendicular laser polarisation (or scanning direction) to the surface roughness directionality, some LIPSS appear aligned with the polishing lines, as could be seen in Fig. 7 at an energy fluence of $2.4\,\text {J/cm}^{e}$, demonstrating that the grooves act as a foundation for LIPSS formation.

## Results of LIPSS formation on the steel surfaces for different scanning directions

Before analysing the measured data obtained from the histogram in the form of colour maps, it is instructive to take a look at the probability distributions of specific LIPSS regularity values R obtained in our experiments. Therefore, we will first compare the observation of the R variance for the two scanning directions studied by emphasising that the orientation of the E-field is always the same as the scanning. Such a comparison is central to the statistical evaluation of scientific data. Then, specific mean values of the measured regularity R are presented in the form of colour maps.

### Conceptualised view

Here, we focus our attention on the numerical parameters that determine the characteristics of the distributions of the measurement results, such as their shape and spread, location of maxima and tails. These steps allow us to exclude the uncertainties in the differences in the estimates of the LIPSS regularities in the cases studied, without having to consider the details of the processes underlying the dispersion of the observations.

**Fig. 8:**
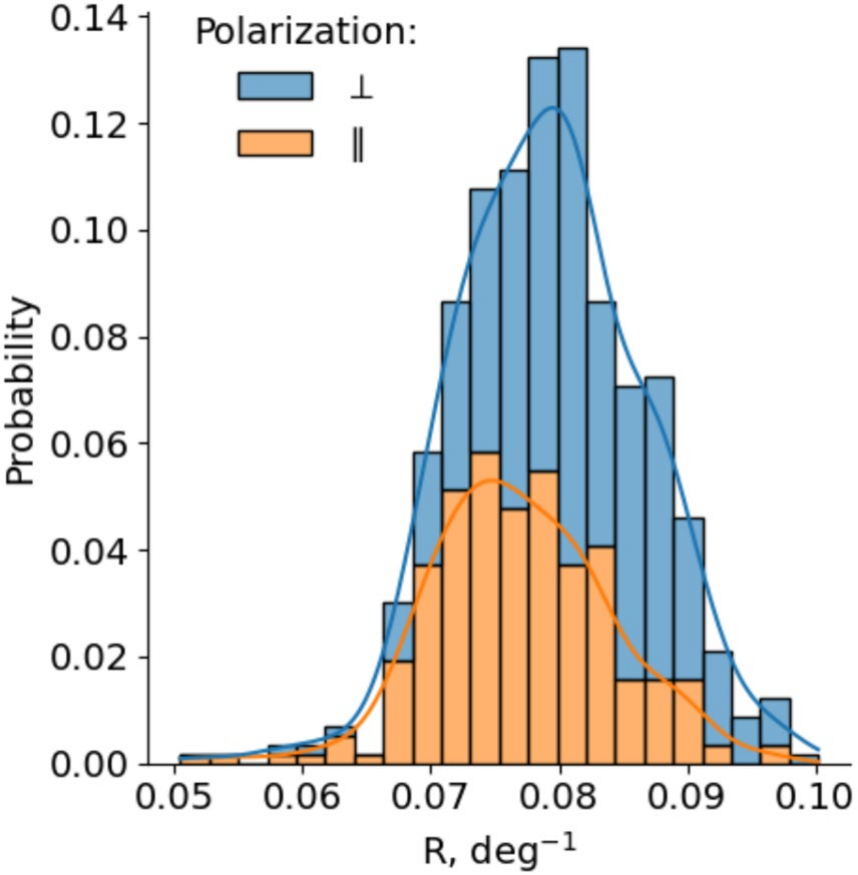
Probability distributions of the regularity parameter *R* for two ($\perp$—perpendicular, and $\parallel$—parallel) polarisation directions of the laser beam.

The Fig. 8 compares the probabilities of specific LIPSS regularity values R when they are formed using different (perpendicular and parallel) directions of laser field polarisation with respect to the dominant surface roughness directionality. Firstly, looking at Fig. 8, it should be emphasised that even though the same number of experiments were carried out in our experiments for both directions, using the same values of beam overlap and power, the total number of LIPSS formed was significantly lower for the parallel direction (i.e. in some cases LIPSS were not even formed, most often, at lower fluence values and lower overlap). In addition, note the pronounced asymmetry in the probability distributions: the distribution of rising fronts (for pure LIPSS regularities) is more or less uniform. Conversely, in the tails of the distributions (with R values approaching 0.1 for DLOA = 10 degrees), there is a much higher probability of HR LIPSS formation when using perpendicular polarisation to the roughness directionality.

Finally, to demonstrate a statistically significant difference between the results obtained in the two directions of field polarisation, we performed a paired *t*-test^34^ between the two groups of measurements and found that *p*=4.64$\cdot$ 10^−5^. Recall that *p*-values are commonly used to test (and dismiss) a ‘null hypothesis’, which generally states that there is no difference between two groups. The smaller the *p*, the less likely an observed set of values would occur by chance, assuming that the null hypothesis is true. The results are considered statistically significant if the *p*-value is less than the generally accepted significance level of 0.05.

### Colour maps of R values obtained using perpendicular and parallel field polarisation with respect to surface roughness directionality

Having noticed that LIPSS possessing higher regularity were formed using beam polarisation perpendicular to the surface roughness directionality, here we compare R values evaluated by averaging R values for each specific measurement at 6 locations on a linearly polished steel surface.

**Fig. 9:**
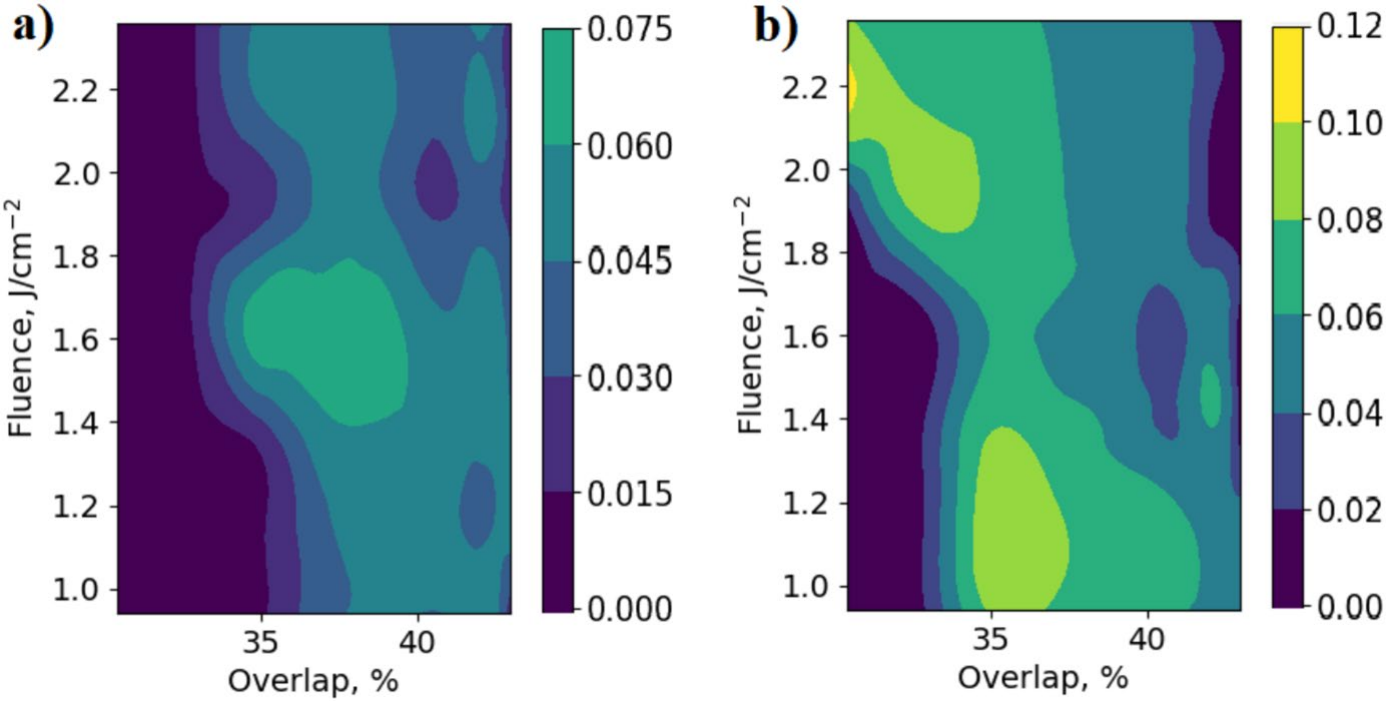
Colour maps of mean LIPSS regularities (R) averaged over 6 measurements at different locations, using a laser beam with parallel (**a**) and perpendicular (**b**) polarisation directions to the initial surface roughness directionality.

The best of R mean values obtained with polarisation being parallel to the polishing grooves as it is shown in Fig. 9a, was 0.72, whereas the best of those obtained with polarisation being perpendicular to the polishing grooves was 1.07 (see Fig. 9b), which is about 50% higher. Notably that here not only the best value of regularity is increased, but the overall average of the values was increased by the same percentage. Here we also see the reduced area of R values in the color map (a) compared to map (b). This observation, that the probability of forming a regular LIPSS is higher when using perpendicular field polarisation with respect to the polished surface grooves, was mentioned above in previous subsection.

It is interesting to note that the locations on the colour maps where the R values are highest are dramatically different. In Fig. 9a they appear to be in the middle, i.e. at the average values of power and overlap used. However, in Fig. 9b they are in opposite corners of the map: top left and bottom middle. Therefore, the technology should take into account that the optimum conditions for LIPSS formation on unpolished and linearly polished surfaces are different.

## Discussions

The above-described femtosecond-laser generated LIPSS are produced for normal incident radiation at laser fluences slightly above the ablation threshold (the ablation threshold for stainless steel with femtosecond lasers is typically in the range of 0.1–0.3 J/cm^2^^35,36^) and have spatial periods close to the laser wavelength $\approx {1}\upmu$m. At our experimental conditions we did not observe any noticeable changes of the period of LIPSS with the polish orientation and fluence/overlap.

The electromagnetic (EM) theory is generally accepted for LSFLs formation. In this theory, optical interference effects resulting from the superposition of incident radiation with electromagnetic waves scattered on a rough surface and propagated very nearly along it, appear as a crucial condition for “classical” LSFLs formation^37^. In order to discuss our results on LIPSS formation using two beam polarisation directions with respect to surface roughness directionality, let us return to the experimental geometry shown in Fig. 2b. Here, the initial linearity of the surface roughness was taken perpendicular and parallel to the *y* (scanning and polarisation) axis, and the schematically depicted LIPSS groves appear perpendicular to the *y*-polarised field. Further, to explain the observed and described above results, two existing theories of light scattering on rough surfaces will be used. It should be noted that both theories are perturbative and analytical expressions used in this Section rely on small surface roughness amplitude: $\sigma<< \lambda$.

### Light scattering at grazing angle

As far as the electromagnetic theory of LSFL formation and its periodicity in metals (often close to the laser wavelength) is based on the interference between the incident laser field and surface plasmon polaritons (SPPs), it is instructive to evaluate and compare the scattered parts of the incident beam at almost grazing angles for parallel and perpendicular directions of beam polarisation to the initial directionality of the surface roughness. Due to the pronounced anisotropy of the surface roughness, the one-dimensional approach (which refers to a surface with variations only along one horizontal coordinate, while keeping constant along the other one) is reasonable for modelling microscopic scattering peculiarities from an initially rough surface.

In the case of a perfectly conducting surface, the normalised intensity dependence of the scattered light intensity on the scattering angles can be written in integral form (see^38,39^), and, at normal incidence, can be expressed as follows:

2 $$\begin{aligned} I(\theta _{o}) &= \int _{0}^{\infty } J_0 \left[ \frac{2\pi y}{\lambda } \sin \theta \right] \nonumber \\ & \quad \times \exp \left[ -(\frac{2\pi \sigma }{\lambda })^2 (1+\cos \theta _o)^2 (1-C(y)) \right] \cdot dy. \end{aligned}$$

Here $J_0$ stands for zero-order Bessel function, *y* is the distance between two points on the surface, $\sigma$ is the RMS roughness (i.e. the root of the mean square deviation of the surface profile), and *C*(*y*)—is the normalised autocorrelation function of the roughness profile of the surface. Equation 2 establishes a relationship between the statistical properties of the surface and the angular scattered intensity.

We have numerically modelled the intensity of the scattering in both directions, using the RMS roughness $\sigma _{\perp ,\parallel }$ = (0.11, 0.04) $\upmu$m and the length of the exponential correlation function $\Lambda _{\perp ,\parallel }$ = (50, 10) $\upmu$m evaluated using the roughness profiles shown in Fig. 5. (The indexes $\perp \text {and} \parallel$ are used here to mark the perpendicular and parallel directions of the field to the linearity of the initial surface roughness, respectively).

**Fig. 10:**
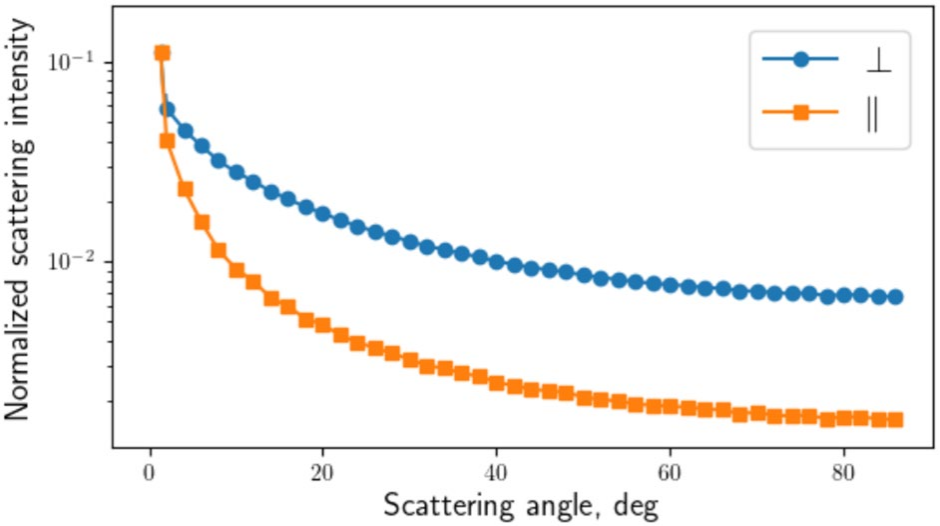
Normalised angular scattered intensity versus scattering angle (degrees) for pairs of the estimated roughness parameters in two directions.

Figure 10 shows the normalised scattered intensities for both cases, for a wavelength of $\lambda =$1.030 $\upmu$m. As noted in^39^, the profile of the angular scattered intensity narrows closer to the specular scattering region as the roughness decreases. Considering our case, in the region of interest (scattering angles close to grazing incidence), the scattered intensity appears to be higher when the scanning beam polarisation is perpendicular to the polished surface linearity.

### Sipe’s efficacy factor theory

The possibility of predicting the appropriate Fourier components of LIPSS on surface roughness and their reinforcement by subsequent laser pulses was demonstrated in^11,37,40–42^. The theory became widely known as Sipe’s efficacy factor theory after the general expressions given in^41^ were simplified and written in a practically convenient form of equations^43^.

The key parameters in this theory are the shape and filling factors *s* and *f*. The best pair of parameters (*s*, *f*) found in the original paper^42^ to describe the experimental formation of LIPSS in Ge is equal to (0.4,0.1). The values found were later used not only for semiconductors, but also for metals and dielectrics (for review see e.g.^44^). The shape factor *s* describes how strongly scattered and incident electromagnetic waves interfere at the surface and is primarily related to its roughness and mathematically can be evaluated as the ratio of the correlation length of the surface roughness to the thickness of the selvedge $\Lambda _S$. As far as evaluated in the previous section, lengths of the correlation functions $\Lambda _{\perp }$ and $\Lambda {\parallel }$ differ, the mathematical expression for the parameter *s* results in a much higher value when the field polarisation vector is perpendicular to the directionality of the surface roughness. Therefore, we will use values $s=1.0$ and $s=0.4$ in our following calculations for perpendicular and parallel orientations, respectively. And the filling factor, which represents the surface fraction filled with scattered islands^41,42^, we will leave unchanged, i.e. $f=0.1$ for both cases of interest.

**Fig. 11:**
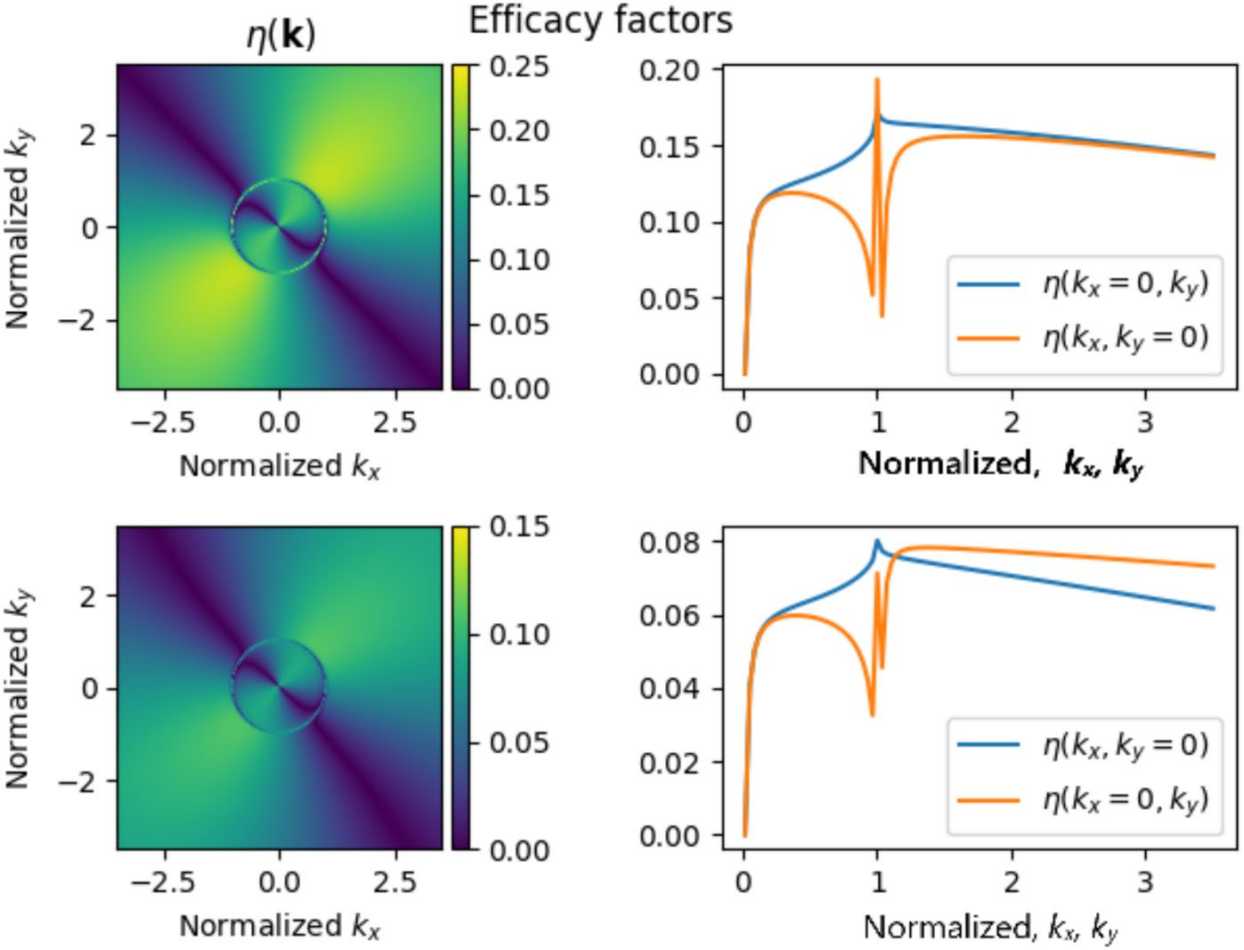
Dependencies of the efficacy factor $\eta$(kx,ky) plotted for the two sample orientations shown in Fig. 2: with field polarisation perpendicular (top row) and parallel (bottom row) to the directionality of the roughness of the surface.

Using the equation from^43^ mentioned above, in Fig. 11 we plot the LIPSS efficacy factor $\eta (kx,ky)$ versus projections (*kx*, *ky*) of the normalised LIPSS wave vector for two scanning cases: with field polarisation perpendicular (top row) and parallel (bottom row) to the directionality of the roughness of the surface. For surface permittivity, the complex value for stainless steel $\epsilon$ = − 13.621 + 39.648i, evaluated from the date base of the optical constants^45^ at 1030 nm, was used.

The solid lines on the graphs in the right column represent the dependencies of the efficacy factor on $k_x$ and $k_y$. The pronounced spike, responsible for the better LIPSS formation efficacy factor with field polarisation being perpendicular to roughness directionality, should be noted in the upper graph.

Finally, it should be noted that Sipe’s theoretical framework of efficiency factors to understand the formation of LIPSS focuses on optical interference patterns and their influence on surface modulation. While the theory provides valuable insights to LIPSS formation, it has some limitations, especially when applied to metals and when multiple laser pulses are used. In this case, the theory would need to be integrated with state-of-the-art models to fully describe the cumulative thermal, hydrodynamic, and plasmonic effects.

## Conclusions

In this study, we investigated the low spatial frequency LIPSS induced on steel samples by a scanning femtosecond laser beam in a series of experiments on the purposely grounded steel surface. A data-driven nonparametric method was used to detect LIPSS regularity based on the estimation of probability density. Our study shows that the probability of higher regularity LIPSS formation is higher when scanning (and maintaining field polarisation) perpendicular to the polishing direction of the surface. The result is not only statistically significant, but also practically significant, as it shows the possibility to improve the formation of LIPSS in the experiment simply by handcrafted polishing of the surface linearly, without any special effort.

Finally, the conclusion would follow that existing electromagnetic theories (Sipe’s efficacy factor theory, e.g.) provide a solid foundation for understanding the formation of LIPSS not only in the cases of single- or low-pulse-number interaction. Electromagnetic theories were shown to be used to evaluate conditions for LIPSS formation even in cases involving multiple-pulse interaction on a large area, where cumulative thermal, hydrodynamic, and plasmonic effects play a significant role, especially for metals.

## Data Availability

Data will be made available on request to the corresponding author.
